# A study on the impact of sports participation support on the level of sports participation of urban junior high school girls in China

**DOI:** 10.3389/fpsyg.2024.1539415

**Published:** 2025-01-28

**Authors:** Mingyuan Dong, Mingtian Niu, Zhengxin Jiang, Yongchul Choi, Ning Li

**Affiliations:** ^1^Department of Physical Education, Gangneung–Wonju National University, Gangneung, Republic of Korea; ^2^Department of Public Physical Education, Xinyang College, Xinyang, China; ^3^School of Computer Science, Wuhan University, Wuhan, China

**Keywords:** physical activity participation support, urban middle school girls, physical activity levels, adolescent girls, sports support systems, youth sports participation

## Abstract

**Background:**

Social support has been identified as a key factor influencing adolescents’ engagement in physical activities. However, the relationship between middle school girls’ physical activity levels and the actual social support they receive remains unclear.

**Aim:**

This study aims to explore the support mechanisms that promote increased physical activity participation among middle school girls.

**Methods:**

A survey was conducted with 1,371 middle school girls from 16 regions and cities in China. The questionnaire included the Physical Activity Questionnaire (PAQ), the Social Support and Exercise Survey, the Physical Activity Rank Scale, and the Family Affluence Scale (FAS). Linear regression was used to examine the relationships among family support, out-of-school exercise behavior, in-school exercise behavior, and the physical activity levels of middle school girls.

**Results:**

In-school physical activity participation (6.6%) was higher than out-of-school participation (5.5%) among urban middle school girls. Physical activity levels were significantly positively correlated with social support from family, peers, and teachers (*p* < 0.01). Linear regression and descriptive statistics consistently showed that family support was the highest (3.50 ± 1.06), followed by teacher support (3.17 ± 0.96) and peer support (2.88 ± 0.96). Emotional support was notably higher, while instrumental support was relatively low. The physical activity levels of urban middle school girls were associated with the age and grade of the individual, the economic conditions of the family, and the highest level of education of the parents.

**Conclusion:**

Family support significantly enhances urban middle school girls’ participation in physical activities. Higher parental education levels may reduce girls’ activity participation, while improved family economic conditions may decrease emotional support but increase informational support. The support of friends and teachers will make up for the decrease in family support. A school-based social support network is essential to address disparities in activity support and implement health intervention programs for middle school girls.

## 1 Introduction

Participation in physical activities among children and adolescents effectively reduces the risk of cardiovascular and other diseases, improves psychological wellbeing, enhances interpersonal communication skills, and significantly boosts cognitive abilities and academic performance. These benefits have been well-documented (Poitras et al., 2016; Donnelly and Lambourne, 2011). However, 81% of adolescents worldwide (aged 11–17 years) fail to meet the recommended levels of physical activity, and participation rates in many countries are declining. Particularly concerning are the participation levels among key demographic groups, such as girls, women, the older adult, and individuals with disabilities (British Heart Foundation, 2015). In 2018, both the WHO in its *Global Action Plan on Physical Activity 2018–2030: More Active People for a Healthier World* and the International Olympic Committee (IOC) in its *Gender Equality and Inclusion Objectives 2021–2024* emphasized the importance of promoting safe, affordable, and accessible opportunities for physical activity through schools, communities, and social policies. These initiatives aim to increase female participation in sports and physical activities, thereby improving health outcomes and fostering social inclusion (World Health Organization, 2024; International Olympic Committee, 2024). Thus, adolescent and female participation in sports has garnered significant societal attention. Adolescent girls, who embody both youth and female identity, represent a key demographic for promoting sports participation. Research indicates that girls’ participation in sports decreases with age. According to WHO recommendations, children and adolescents should engage in at least 1 h of moderate-to-vigorous physical activity daily. Yet, most girls fail to meet this standard. In China, only 30% of school-aged children met the activity guidelines in 2016, with boys at 32% and girls at a mere 28% (Fan and Cao, 2017). Similarly, a decade-long study by the Tufts University John Hancock Research Center on Physical Activity, Nutrition, and Obesity Prevention in the United States revealed that while 57.8% of boys met the recommended activity levels, only 39.1% of girls did so, with a mere 3% of girls aged 12–15 meeting the daily exercise standard (The 2018 Tucker Center Research Report, 2018). Both China and other developed nations face declining sports participation rates among adolescent girls, particularly during early adolescence (middle school years).

Academic research on promoting female sports participation often focuses on social support. The social networks formed by girls with friends, family, and teachers are closely tied to their involvement in physical activities (The 2018 Tucker Center Research Report, 2018)^.^ Many scholars employ a socio-ecological framework to analyze individual, interpersonal, environmental, and policy factors affecting sports participation. Factors such as age, gender, health status, self-efficacy, and other motivational and emotional variables are all related to participation. However, fewer studies have been conducted on the interpersonal perspective (Mendonca et al., 2014; Casey et al., 2009; Craike et al., 2009).

Human behavior is inherently social, and health behaviors often reflect those of one’s social connections. Individuals who rarely engage in physical activities tend to associate with others who also lack participation, leading to fewer opportunities and lower levels of social support (Mötteli and Dohle, 2020). Family, peer, school, and community support all influence adolescents’ physical activity behaviors to varying degrees (Morrissey et al., 2015). Reimers et al. (2019) found a correlation between physical activity and social support from parents and peers, with differences noted between boys and girls. Edwardson et al. (2013) further noted that boys were more likely than girls to perceive peer support for physical activities. For adolescent girls, research often centers on parental and peer support but overlooks the broader social network or the role of teachers. A lack of technical competence among physical education teachers to meet girls’ needs, combined with gender-biased attitudes favoring boys, can deter girls’ participation (Yao and Rhodes, 2015; Carlin et al., 2018; Davison, 2004). Parents are also more likely to encourage boys to engage in sports activities compared to girls (Reimers et al., 2019). Laird et al. (2018) interviewed girls aged 13–15 and found that unfamiliarity with the environment or activities, coupled with fear of being ridiculed, discouraged participation. However, encouragement from family and friends helped alleviate psychological barriers, enabling girls to engage in sports. Moreover, without financial support from parents, girls face difficulties accessing sports activities. Parental and peer support is crucial for adolescent girls, with parents serving as role models who subtly influence their daughters’ participation in sports.

Social support is defined as resources provided through interactions with significant others (e.g., parents, peers) within a social network, which can influence behaviors (Langford et al., 1997; Sheridan and Radmacher, 1992). Specific connections within social networks can impact sports participation through various mechanisms (Social networks in urban situations, 1969). For middle school girls, one critical mechanism is social support, which may take the form of emotional (e.g., encouragement, praise), instrumental (e.g., provision of equipment or financial aid), or informational support (e.g., advice, guidance). These supports are often provided by individuals within the girls’ social network, such as friends, family, or teachers (Laird et al., 2018). Researchers have categorized social support into tangible (e.g., financial aid, transportation) and intangible forms (e.g., praise, encouragement). However, it remains unclear which specific behaviors most effectively promote adolescent sports participation, necessitating a more comprehensive evaluation of support factors (Yao and Rhodes, 2015). Four types of social support can be summarized: (1) Emotional Support: Providing intangible resources such as love, empathy, care, and trust. (2) Instrumental Support: Offering tangible resources or services directly beneficial to the recipient (e.g., money, transportation, or access to sports facilities). (3) Informational Support: Helping recipients solve problems by providing information, advice, or guidance (e.g., explaining the benefits of physical activity). (4) Appraisal Support: Offering constructive feedback, positive comparisons, or encouragement to help recipients assess self-efficacy or competence (Donev et al., 2007; Deci and Ryan, 2013). Based on these insights, this study examines the effects of emotional, instrumental, informational, and appraisal support from family, peers, and teachers on girls’ sports participation behaviors. In the context of the urgent need to improve the physical fitness of adolescents, the study of the physical activity level of urban junior high school girls is of great significance. Through the comparison of relevant studies, it is found that the relationship between social support and the promotion of junior high school girls’ sports participation is not very clear. Therefore, this study takes Chinese urban junior high school girls as the survey subjects and focuses on the impact of social support on girls’ sports participation behavior.

## 2 Methods

### 2.1 Research object

After the initial questionnaire design was completed, 10 field experts with high relevance to the topic were invited to evaluate its validity. Among them, 40% deemed the content highly reasonable, while 60% found it fairly reasonable. Regarding structural evaluation, 20% rated it as highly reasonable, and 80% considered it fairly reasonable. Based on expert feedback, the questionnaire was revised and piloted in two middle schools in City B, Henan Province. A total of 98 questionnaires were distributed, during which the students’ real-time experiences and reactions while completing the questionnaire were observed and recorded to assess its readability.

The survey targeted urban middle school girls in grades 7–9 across China. Using stratified sampling, survey cities were selected based on the regional division of China’s eastern, central, western, and northeastern areas as defined by the National Bureau of Statistics. The selected cities included: (1) Central China: Zhengzhou (Henan), Changsha (Hunan); (2) East China: Wuxi (Jiangsu), Linyi (Shandong), Hefei (Anhui), Quanzhou (Fujian), Shanghai; (3) South China: Guangzhou, Shenzhen (Guangdong), Qinzhou (Guangxi); (4) North China: Tianjin, Beijing, Chengde (Hebei), Changzhi (Shanxi); (5) Northeast China: Shenyang (Liaoning). For representativeness, one public and one private school were selected from each city. Within each school, one class per grade was randomly chosen. To ensure participants’ accessibility, a collective paper-and-pencil approach was adopted under the supervision of homeroom teachers, with at least 10 min allocated for questionnaire completion. A total of 1,630 questionnaires were distributed, 1,535 were returned (response rate: 94.17%), and 1,371 valid responses were included (validity rate: 89.32%).

#### 2.1.1 Dependent variable

The Physical Activity Questionnaire (PAQ), a self-administered 7-day recall tool, was used to assess the overall level of physical activity in students across various age groups. It evaluates participation in different types of activities over the past week, as well as self-perceived activity levels, with the average score representing participation levels (Kowalski et al., 2004). For middle school girls, a subset of representative items from PAQ was utilized. Using a 5-point Likert scale, participants rated their activity levels, where higher scores indicated greater participation (maximum: 5; minimum: 1), the questionnaire contained seven items. K-S normality test: Significant (*p* < 0.05, df = 6); Exploratory factor analysis: KMO = 0.820, Bartlett’s test: *χ*^2^ = 4017.556, df = 21, *p* < 0.001; Factor variance: 0.532–0.756; Cronbach’s *α* = 0.835; split-half reliability = 0.670.

#### 2.1.2 Independent variables

The Social Support and Exercise Survey was adapted to measure support for physical activity from family, friends, and peers (Barber, 2012). The design of the social support scale was based on social support network theory (Laird et al., 2016) and modified from the original Social Support and Exercise Survey (Sarason et al., 1983). It assessed support through four dimensions—emotional, instrumental, informational, and appraisal support—on a 5-point Likert scale, where higher scores reflected stronger support. The scale comprised 36 items across three categories (family, friends, teachers, with 12 items each). Social Support Scale: K-S normality test: Significant (*p* < 0.05, df = 35); Exploratory factor analysis: KMO = 0.950, Bartlett’s test: *χ*^2^ = 37192.222, df = 630, *p* < 0.001; Factor variance: 0.532–0.756; Cronbach’s *α* = 0.955; split-half reliability = 0.857.

Key dimensions were individually analyzed: (1) Family Support: K-S test: Significant (*p* < 0.05, df = 11); KMO = 0.944, Bartlett’s test: *χ*^2^ = 11693.159, df = 66, *p* < 0.001; Factor variance: 0.479–0.692; Cronbach’s *α* = 0.940; split-half reliability = 0.885. (2) Friend Support: K-S test: Significant (*p* < 0.05, df = 11); KMO = 0.923, Bartlett’s test: *χ*^2^ = 10521.082, df = 66, *p* < 0.001; Factor variance: 0.585–0.788; Cronbach’s *α* = 0.916; split-half reliability = 0.830. (3) Teacher Support: K-S test: Significant (*p* < 0.05, df = 11); KMO = 0.921, Bartlett’s test: *χ*^2^ = 10465.422, df = 66, *p* < 0.001; Factor variance: 0.610–0.767; Cronbach’s *α* = 0.910; split-half reliability = 0.809.

In 2010, the WHO issued its Global Recommendations on Physical Activity for Health, advocating that children and adolescents aged 5–17 engage in at least 60 min of moderate-to-vigorous intensity physical activity daily (World Health Organization, 2010). This study adopts the WHO’s criteria, defining junior high school girls who participate in 60 min or more of moderate or vigorous physical activity each day as meeting the exercise standard (henceforth referred to as “meeting the exercise standard”). Consequently, a questionnaire survey was conducted to assess the daily physical exercise status of junior high school girls, categorizing them as either not meeting (0- or meeting; 1- the exercise standard).

#### 2.1.3 Control variables

Control variables included age, grade, parental education, and family wealth status, which were encoded as follows: Age (1 = below 12, 2 = 12, 3 = 13, 4 = 14, 5 = 15, 6 = 16).Grade: (1 = 7th grade, 2 = 8th grade, 3 = 9th grade).Parental Education: (1 = junior high school or below, 2 = high school/vocational school, 3 = associate degree, 4 = bachelor’s degree, 5 = master’s degree or above).Family Wealth Status: (0 = economically disadvantaged, 1 = economically advantaged).

The Family Affluence Scale (FAS) (Torsheim et al., 2016) was adapted for assessing socio-economic status. Four items were selected from the original 16 to represent middle schoolers’ family conditions: parental employment, owning a private bedroom, car ownership, and computer ownership. Affluence was classified as: Disadvantaged-Parents unemployed, no car, no private bedroom, no computer. Advantaged-At least one parent employed, at least one car, a private bedroom, and at least one computer.

### 2.2 Analytical methodology

Multiple linear regression was used to analyze the relationship between urban middle school girls’ physical activity levels, different dimensions of social support, and adherence to exercise standards both in and outside school. The weekly average physical activity score was treated as the dependent variable, while dimensions of social support and exercise adherence served as independent variables. Grade, parental education, and family wealth were included as control variables in the regression model.

## 3 Results

### 3.1 Basic characteristics of the sample

The characteristics of the sample (Table 1) indicate that most urban middle school girls are of Han ethnicity, with ages ranging from 12 to 15 years. The highest educational attainment of their parents predominantly falls into three categories: high school/vocational school, associate degree, and bachelor’s degree. Approximately 70% of urban middle school girls come from families with favorable economic conditions. However, only 6.6% of these girls met the recommended daily standard of 1 h of moderate-to-vigorous physical activity within school, and just 5.5% did so outside school, suggesting that participation in physical activities is notably higher within school settings. Overall, the social support for physical activity among urban middle school girls is ranked as follows: family support (3.50 ± 1.06) > teacher support (3.17 ± 0.96) > peer support (2.88 ± 0.96). Among the dimensions of support, emotional support is the highest, whereas instrumental support is the lowest. Emotional support mainly encompasses encouragement from family, peers, and teachers for engaging in physical activities. In contrast, instrumental support includes financial assistance, provision of sports equipment, and the organization of leisure-time activities that promote participation in physical exercises.

**Table 1:** Characteristics of the study sample (*N* = 1,371).

		*N*	%
Ethnic group	Han ethnicity	1,313	95.8
	Minority ethnicity	58	4.2
Age	1 = below 12	33	2.4
	2 = 12	222	16.2
	3 = 13	570	41.6
	4 = 14	364	26.5
	5 = 15	154	11.2
	6 = 16	28	2.1
Grade	1 = 7th grade	532	38.8
	2 = 8th grade	565	41.2
	3 = 9th grade	274	20
Parent’s highest education level	1 = Junior high school and below	176	12.8
	2 = high school/vocational school	485	35.4
	3 = associate degree	219	16
	4 = bachelor’s degree	315	23
	5 = Master degree or above	176	12.8
Family economic status	0 = Low-income family	411	30
	1 = High-income family	960	70
Daily exercise on campus	1 = Meets exercise standards	90	6.6
	0 = Does not meet exercise standards	1,281	93.4
Daily exercise off campus	1 = Meets exercise standards	75	5.5
	0 = Does not meet exercise standards	1,296	94.5

	Mean	SD
Past week exercise activity	3.05	0.78
Average family social support	3.5	1.06
Average family emotional support	3.71	1.12
Average family instrumental support	3.45	1.19
Average family informational support	3.48	1.18
Average family evaluative support	3.28	1.33
Average friend social support	2.88	0.96
Average friend emotional support	3.36	1.15
Average friend instrumental support	2.4	1
Average friend informational support	3.01	1.2
Average friend evaluative support	2.9	1.28
Average teacher social support	3.17	0.96
Average teacher emotional support	3.56	1.16
Average teacher instrumental support	2.53	1.01
Average teacher informational support	3.48	1.18
Average teacher evaluative support	3.38	1.32

### 3.2 Correlation analysis of physical activity participation and support

There is a significant positive correlation between the level of physical activity participation and support from family, peers, and teachers (Table 2). The correlation coefficient between physical activity participation and emotional support from family is 0.242, whereas the correlation coefficient with instrumental support from teachers is relatively smaller (0.142). Nevertheless, the findings affirm that higher levels of social support from family, peers, and teachers correspond to greater participation in physical activities among urban middle school girls. These results provide a basis for further stepwise regression analysis to identify the relationships between physical activity participation and support from these groups.

**Table 2:** Correlation analysis between sports participation and social support for junior high school girls.

	A	J1	J2	J3	J4	P1	P2	P3	P4	S1	S2	S3	S4
A	1												
J1	0.242**	1											
J2	0.200**	0.722**	1										
J3	0.217**	0.463**	0.456**	1									
J4	0.204**	0.637**	0.670**	0.398**	1								
P1	0.220**	0.554**	0.452**	0.499**	0.411**	1							
P2	0.137**	0.263**	0.350**	0.308**	0.328**	0.455**	1						
P3	0.190**	0.417**	0.444**	0.546**	0.471**	0.718**	0.597**	1					
P4	0.205**	0.375**	0.400**	0.457**	0.503**	0.622**	0.537**	0.812**	1				
S1	0.216**	0.594**	0.484**	0.760**	0.421**	0.604**	0.278**	0.490**	0.431**	1			
S2	0.142**	0.291**	0.348**	0.517**	0.313**	0.324**	0.582**	0.402**	0.371**	0.459**	1		
S3	0.217**	0.463**	0.456**	1.000**	0.398**	0.499**	0.308**	0.546**	0.457**	0.760**	0.517**	1	
S4	0.193**	0.401**	0.427**	0.837**	0.400**	0.448**	0.295**	0.499**	0.477**	0.671**	0.480**	0.837**	1

**p < 0.01, **p < 0.05, significant correlation (A: Average physical activity over the past 7 days; J1: Average emotional support from family; J2: Average instrumental support from family; J3: Average informational support from family; J4: Average evaluative support from family; P1: Average emotional support from friends; P2: Average instrumental support from friends; P3: Average informational support from friends; P4: Average evaluative support from friends; S1: Average emotional support from teachers; S2: Average instrumental support from teachers; S3: Average informational support from teachers; S4: Average evaluative support from teachers).

### 3.3 Regression analysis of physical activity participation and support

#### 3.3.1 Differences in support by grade and age

Using grade as a grouping variable, three regression models were developed (Table 3). The results indicate significant relationships between physical activity participation and family support (emotional, informational, and evaluative support), peer support (emotional and evaluative support), control variables (parental education and age), and meeting physical activity standards within school settings. Support from family was more influential for 7th-grade girls, with a one-unit increase in evaluative support leading to a 12.1% increase in their physical activity level. For 8th-grade girls, emotional support from family had the greatest impact, with a one-unit increase leading to a 6.9% increase in physical activity level. For 9th-grade girls, emotional support from peers was most impactful, with a one-unit increase resulting in an 11.2% increase in activity level. Among the control variables, age had a moderating effect on 9th-grade girls, with older students showing higher levels of physical activity. Conversely, higher parental education was negatively correlated with the activity levels of 9th-grade girls, with each additional level of parental education associated with a 7.1% decrease in activity. For girls meeting physical activity standards within schools, 9th-grade students exhibited the largest increase in participation levels (85.3%). Teacher support showed no significant effect across grades, indicating no grade-related differences in its impact.

**Table 3:** Regression models of physical activity involvement and social support among urban junior high school girls by grade level.

	7th grade	8th grade	9th grade
Family support			
Family emotional support		0.069^*^ (0.031)	
Family informational support	0.114^**^ (0.030)		0.085^*^ (0.040)
Family evaluative support	0.121^**^ (0.026)		
Friend support			
Friend emotional support			0.112^*^ (0.046)
Friend evaluative support		0.094^**^ (0.027)	
Control variables			
Parents’ highest education level			−0.071^*^ (0.032)
Age			0.157^*^ (0.046)
Meeting exercise standards			
On-campus activities meet the standards	0.343^*^ (0.154)	0.410^**^ (0.117)	0.853^**^ (0.154)
Constant	2.210^**^ (0.106)	2.484^**^ (0.120)	1.503^**^ (0.277)
*R* ^2^	0.119	0.063	0.286

**p < 0.01, **p < 0.05, values in parentheses are standard deviations.

Using age as a grouping variable, six regression models were constructed (Table 4). The results reveal significant relationships between physical activity participation and family support (emotional, instrumental, and informational support), peer support (emotional and evaluative support), teacher support (instrumental and evaluative support), control variables (parental education), and meeting physical activity standards (both within and outside school).

**Table 4:** Regression models of physical activity involvement and social support for urban junior high school girls by age groups.

	Under 12 years old	12	13	14	15	16
Family support						
Family emotional support			0.137^**^ (0.033)		0.149^*^ (0.059)	
Family instrumental support					0.106^*^ (0.053)	
Family informational support		0.185^**^ (0.054)				
Friend support						
Friend emotional support			0.071^*^ (0.032)			
Friend evaluative support				0.151^**^ (0.029)		
Teacher support						
Teacher instrumental support		0.159^**^ (0.060)				0.317^*^ (0.151)
Teacher evaluative support	0.437^**^ (0.084)					
Control variables						
Highest parental education level					−0.148^**^ (0.047)	
Meets exercise standards						
Compliant with on-campus standards			0.415^**^ (0.125)	0.637^**^ (0.129)	1.011^**^ (0.268)	2.169^**^ (0.621)
Compliant with off-campus standards	−0.874^*^ (0.389)					
Constant	1.924^**^ (0.258)	1.983^**^ (0.172)	2.264^**^ (0.115)	2.531^**^ (0.094)	2.598^**^ (0.247)	2.355^**^ (0.367)
*R* ^2^	0.526	0.151	0.095	0.125	0.271	0.368

**p < 0.01, **p < 0.05, values in parentheses are standard deviations.

The findings highlight that informational support from family was particularly impactful for 12-year-old girls (18.5%), whereas emotional and instrumental support from family were more influential for 15-year-old girls (14.9 and 10.6%, respectively). Emotional support from peers significantly enhanced the activity levels of 13-year-old girls (7.1%), while evaluative support from peers was most beneficial for 14-year-old girls (15.1%). Instrumental support from teachers notably increased the activity levels of 16-year-old girls (31.7%), and evaluative support from teachers had the highest impact on girls under 12 years old (43.7%). Among control variables, higher parental education levels were negatively correlated with the activity levels of 15-year-old girls, with each additional level of education associated with a 14.8% decrease. For 15- and 16-year-old girls meeting physical activity standards within school, activity levels increased by over 100% per unit increase. However, for girls under 12, meeting the standards outside school corresponded to an 87.4% decrease in overall activity levels, suggesting that out-of-school activities were less effective in promoting physical activity among this age group.

#### 3.3.2 Differences in support by family context

Using parental education as a grouping variable, five regression models were developed (Table 5). The results show significant positive correlations between physical activity participation and family support (emotional, informational, and evaluative support), peer support (emotional and informational support), control variables (grade), and meeting physical activity standards (within and outside school). Emotional support from family had the greatest impact on girls whose parents had a bachelor’s degree (12.3%). Informational and evaluative support from family also significantly increased activity levels for girls with parents holding a bachelor’s degree (16.4 and 13.0%, respectively). Emotional support from peers was most beneficial for girls with parents holding a graduate degree or higher (15.0%), while informational support from peers was most impactful for girls with parents having a high school/vocational school education (6.5%). Among control variables, grade had the greatest positive effect on activity levels for girls with parents holding a high school/vocational school education (8.9%). Meeting physical activity standards within school was particularly impactful for girls whose parents had less than a junior high school education, increasing activity levels by 74.1%. Meeting standards outside school significantly boosted activity levels for girls with parents having a high school/vocational education (62.0%).

**Table 5:** Regression model of physical activity participation and social support among urban junior high school girls by parental highest education level.

	Junior high school and below	High school/Vocational school	Associate degree	Bachelor’s degree	Master degree or above
Family support					
Family emotional support		0.112^**^ (0.029)		0.123^*^ (0.050)	
Family informational support	0.131^**^ (0.049)		0.164^**^ (0.048)		
Family evaluative support			0.130^**^ (0.040)		
Friend support					
Friend emotional support	0.141^**^ (0.049)			0.147^**^ (0.043)	0.150^**^ (0.057)
Friend informational support		0.065^*^ (0.027)			
Control variables					
Grade		0.089^*^ (0.040)			
Meets exercise standards					
Compliant with on-campus standards	0.741^**^ (0.239)			0.546^**^ (0.167)	0.582^**^ (0.212)
Compliant with off-campus standards		0.620^**^ (0.151)			
Constant	2.143^**^ (0.183)	2.263^**^ (0.125)	2.057^**^ (0.176)	1.964^**^ (0.179)	2.483^**^ (0.210)
*R* ^2^	0.183	0.117	0.142	0.129	0.078

**p < 0.01, **p < 0.05, values in parentheses are standard deviations.

Using family economic conditions as a grouping variable, two regression models were constructed (Table 6). The results indicate significant positive correlations between physical activity participation and family support (emotional and informational support), peer support (evaluative support), and meeting physical activity standards (within school). Emotional support from family had the greatest impact on girls from less affluent families (15.9%), while informational support was more impactful for girls from affluent families (6.3%). Evaluative support from peers significantly enhanced activity levels for girls from affluent families (9.6%), while instrumental support from teachers was more beneficial for girls from less affluent families (7.6%). Meeting physical activity standards within school had the largest positive effect on girls from less affluent families (58.1%). The regression models show minimal moderating effects of control variables (age, grade, and parental education) on the relationship between economic conditions and activity levels.

**Table 6:** Regression model of physical activity participation and social support in urban junior high school girls by family economic status.

	Low-income family	Affluent family
Family support		
Family emotional support	0.159^**^ (0.032)	0.083^**^ (0.025)
Family informational support		0.063^*^ (0.025)
Friend support		
Friend evaluative support		0.096^**^ (0.022)
Teacher support		
Teacher instrumental support	0.076^*^ (0.038)	
Meets exercise standards		
Compliant with on-campus standards	0.581^**^ (0.162)	0.467^**^ (0.093)
Constant	2.294^**^ (0.130)	2.178^**^ (0.095)
*R* ^2^	0.111	0.113

**p < 0.01, **p < 0.05, values in parentheses are standard deviations.

Through the model analysis, it is found that the participation level of urban junior middle school girls in sports activities has nothing to do with her nationality, but is related to her age, grade, family economic conditions, and the highest educational level of her parents, and there is a significant correlation with in-school and out-of-school sports participation meeting the exercise standards. At the same time, sports participation support can significantly improve the participation level of urban junior middle school girls.

## 4 Discussion

### 4.1 Variability in basic factors

The increased weighting of physical education (PE) scores in China’s high school entrance examination has set higher expectations for urban junior high school girls to participate in physical activities (Dong et al., 2023). This study revealed a significant positive correlation between individual characteristics (age and grade) and the level of physical activity participation among urban junior high school girls. Advancing age significantly improved physical activity participation levels among higher-grade (Grade 9) urban girls. Similarly, grade advancement positively influenced participation levels. Age and grade mutually influenced each other, as girls aged and advanced through grades, the emphasis on PE due to national exam policies and local development strategies contributed to improved participation. In contrast, family circumstances, specifically parents’ highest educational attainment, showed a significant negative correlation with activity levels. Higher parental education was associated with reduced physical activity participation, particularly among older girls (15 years) in higher grades. Recent research (Muñoz-Comet and Martínez-Pastor, 2021) highlighted disparities in children’s sports participation based on parental social class, with children of non-upper-class parents engaging more in sports. This suggests that higher parental education does not necessarily enhance girls’ physical activity levels.

Ensuring at least one hour of daily exercise both in and out of school has become a priority in China. Compliance with exercise standards, both on- and off-campus, significantly correlates with urban junior high school girls’ overall physical activity levels. The study found a strong positive correlation between on-campus exercise compliance and participation levels. On-campus compliance significantly improved activity levels among higher-grade girls, older participants (15–16 years), those with higher parental education (graduate or above), and girls from economically poor families. As girls advance in age and grade, increased on-campus exercise due to impending PE exams is inevitable. WHO recommends at least 60 min of moderate-to-vigorous physical activity daily for children and adolescents aged 5–17 (World Health Organization, 2010). However, the proportion of girls meeting exercise standards remains low both on-campus (6.6%) and off-campus (5.5%) in urban areas. Off-campus compliance only improved activity levels for girls whose parents had lower educational attainment (high school/vocational school) and decreased levels for younger girls (below 12 years). Compared to on-campus compliance, off-campus compliance had a smaller or even negative effect on participation levels for some groups. Overall, integrating on- and off-campus exercise is critical to enhancing urban junior high school girls’ activity levels. While school-based programs play a pivotal role, family and community initiatives are equally essential. A school-centered “family-school-community collaboration” model is recommended to synergistically improve both on- and off-campus activity levels among girls.

In addition, when designing school-based and external physical activity intervention programs, it is essential to place significant emphasis on ethical considerations. For instance, the study by Lysdahl et al. (2016) highlights the importance of ethical analysis in complex health interventions, which involves addressing the needs and expectations of participants. Therefore, when developing school-supported networks, ethical factors must be carefully taken into account, and corresponding ethical analysis methods should be employed to ensure the rationality and feasibility of the programs. This participatory research approach provides children with opportunities to engage in the process of knowledge generation. However, it also requires researchers to fully consider the characteristics and needs of girls during the design and implementation of the study. Appropriate strategies must be adopted to balance the research objectives with the rights of the girls, ensuring their genuine participation under conditions that respect their wishes and capabilities. Similarly, the views of Minacori et al. underscore the governance role of research ethics committees, which ensure that clinical research complies with ethical standards and protects the rights of participants (Minacori et al., 2015). This structured intervention approach is equally applicable to adolescent girls’ physical activity intervention programs, as it plays a critical role in ensuring the quality and safety of research initiatives.

### 4.2 Analysis of differences in support for sports participation

From the grade-based perspective, lower-grade (Grade 7) girls rely primarily on family support (informational and evaluative support) to improve participation levels. For middle- and higher-grade girls (Grades 8–9), friend support (emotional and evaluative) supplements family support (emotional and informational). Feedback and analysis of physical activity from family members was a key motivator for younger girls to start physical activity. After a year of school, stronger friendships emerge, and peer evaluative influence becomes more significant. For this age group, positive feedback from friends drives participation. Previous research has also confirmed that the support of friends can maintain the level of physical activity among teenagers (Davison, 2004), and can also promote the motivation of teenagers to persist in physical exercise (Zou et al., 2023), with emotional support being especially influential for older girls.

From an age-based perspective, middle-aged girls (13–15 years) show similar reliance on family and peer support to enhance participation. Family instrumental support includes providing resources such as sports gear, financial assistance, transportation, or organizing activities. Informational support, like sharing benefits and tips about exercise, further encourages participation, particularly for lower-grade girls unfamiliar with sports. For higher-grade girls preparing for PE exams, families’ emphasis on performance boosts activity levels. Key motivations for family encouragement include improving athletic skills (73.3%), health (76.7%), and preparing for mandatory PE exams (49.5%). For urban middle school girls aged 13–15 years, teacher support plays a critical role in promoting physical activity participation, consistent with recent research findings. Studies have shown a significant correlation between teacher involvement and students’ autonomous participation in physical activities, though these studies did not conduct detailed analyses based on grade levels (de Oliveira et al., 2024). The study reveals that teacher support—including instrumental and evaluative support—has a stronger influence on younger (below 12 years) and older (12 and 16 years) girls. Younger girls, due to their relatively underdeveloped physical and psychological maturity, are more reliant on school physical education teachers for assistance. For example, guidance and feedback from physical education teachers during classes are particularly effective in helping girls at this age build self-confidence.

From the model of the highest educational level of parents of urban junior high school girls, girls with parents holding undergraduate degrees or below benefit from both family (emotional, informational, and evaluative) and peer support. For those with highly educated parents (graduate or above), only peer emotional support significantly influences participation. Higher parental education correlates with reduced family support, placing greater reliance on peer encouragement. Family economic status also shapes support dynamics. This is consistent with recent findings that higher family socioeconomic status is positively associated with higher levels of physical activity in adolescents (Craggs et al., 2011). Girls from economically disadvantaged families depend more on family emotional and teacher instrumental support, while those from affluent families benefit from family informational and peer evaluative support. Disadvantaged families often provide higher emotional support, whereas wealthier families emphasize informational support due to better access to sports-related resources. Physical activity promotes educational equity by enhancing cognitive and academic performance in lower-income families, while its effect on higher-income families is negligible (Donnelly and Lambourne, 2011). Thus, while wealthier families provide informational advantages, emotional support remains more pronounced in disadvantaged households.

In summary, parental support is significantly associated with girls’ participation in physical activities (Yao and Rhodes, 2015). Support from the family plays a crucial role in promoting and sustaining adolescents’ physical activity levels (Davison, 2004). Social network support from family members can effectively enhance urban middle school girls’ engagement in physical activities, with research demonstrating that parental support substantially improves their participation levels. Social support is a key factor in enhancing girls’ self-efficacy in physical activity and is strongly correlated with their participation levels (Laird et al., 2018). It helps girls build self-efficacy, enabling them to overcome challenges encountered during physical activities. Support from family, peers, and teachers is particularly beneficial in encouraging girls to participate in sports and exercise (Standiford, 2013).

## 5 Conclusion

The results indicate that urban middle school girls exhibit higher levels of participation in school-based physical activities compared to out-of-school activities. Physical activity participation is significantly and positively correlated with social support. Among various types of support, emotional support is more prevalent, whereas instrumental support is relatively lacking; family support is stronger than teacher support.

The role of social support in enhancing self-efficacy among middle school girls cannot be underestimated. Both intangible and tangible forms of support are vital, with family playing an indispensable role in the girls’ participation in physical activities. Family support significantly increases the level of physical activity participation among urban middle school girls. However, within family support, higher parental educational attainment may lead to decreased participation levels, while better family economic conditions might result in reduced emotional support but increased informational support. When family support diminishes, peer and teacher support often compensate for this gap.

To address the disparities in support for physical activity participation and the differences in school-based and out-of-school participation levels, a “family-school-community” co-education system should be established and refined. This system should build a social support network rooted in schools to promote the implementation of physical fitness and health intervention programs. Specific measures could include: (1) Collaborating with schools, families, and communities: Conduct seminars on sports and health to disseminate knowledge, teach students and their families how to organize physical activities during leisure time, and provide borrowing or renting services for sports venues and equipment. These initiatives can help girls and their peers address the lack of instrumental and informational support. (2) Introducing family-based sports assignments: Encourage parents to supervise students’ participation in out-of-school physical activities, offering emotional support and logistical assistance. (3) Promoting physical education curriculum reform: Conduct regular training for school physical education teachers to enhance their ability to provide support. This includes improving teachers’ understanding of emotional encouragement and informational support for girls’ participation in sports.

The authors acknowledge several limitations in the present study. First, the study design did not adequately differentiate the sources of social support, such as those from peers, family members, and teachers. As a result, the analysis showed limited significance for certain social support variables, particularly those related to peers and teachers. Second, the reliance on self-reported measures of interpersonal support introduces potential biases and restricts the scope of the findings. Future studies could benefit from incorporating objective measures of support, including those derived from the social environment, built environment, social media, and broader societal beliefs. Lastly, this study did not specifically examine the participation levels or influencing factors among rural middle school girls, which limits the generalizability of the findings. Future research should address this gap to provide a more comprehensive understanding of the factors influencing physical activity participation among Chinese middle school girls.

## Data Availability

The raw data supporting the conclusions of this article will be made available by the authors, without undue reservation.
